# Predicting the clinical prognosis of acute ischemic stroke using machine learning: an application of radiomic biomarkers on non-contrast CT after intravascular interventional treatment

**DOI:** 10.3389/fninf.2024.1400702

**Published:** 2024-08-22

**Authors:** Hongxian Gu, Yuting Yan, Xiaodong He, Yuyun Xu, Yuguo Wei, Yuan Shao

**Affiliations:** ^1^Department of Radiology, The People's Hospital of Jianyang City, Jianyang, Sichuan Province, China; ^2^Center for Rehabilitation Medicine, Department of Radiology, Zhejiang Provincial People's Hospital, Hangzhou, Zhejiang, China; ^3^GE Healthcare Life Sciences, Hangzhou, Jiangsu, China

**Keywords:** acute ischemic stroke, machine learning, radiomics signature, computed tomography, stroke—diagnosis

## Abstract

**Purpose:**

This study aimed to develop a radiomic model based on non-contrast computed tomography (NCCT) after interventional treatment to predict the clinical prognosis of acute ischemic stroke (AIS) with large vessel occlusion.

**Methods:**

We retrospectively collected 141 cases of AIS from 2016 to 2020 and analyzed the patients' clinical data as well as NCCT data after interventional treatment. Then, the total dataset was divided into training and testing sets according to the subject serial number. The cerebral hemispheres on the infarct side were segmented for radiomics signature extraction. After radiomics signatures were standardized and dimensionality reduced, the training set was used to construct a radiomics model using machine learning. The testing set was then used to validate the prediction model, which was evaluated based on discrimination, calibration, and clinical utility. Finally, a joint model was constructed by incorporating the radiomics signatures and clinical data.

**Results:**

The AUCs of the joint model, radiomics signature, NIHSS score, and hypertension were 0.900, 0.863, 0.727, and 0.591, respectively, in the training set. In the testing set, the AUCs of the joint model, radiomics signature, NIHSS score, and hypertension were 0.885, 0.840, 0.721, and 0.590, respectively.

**Conclusion:**

Our results provided evidence that using post-interventional NCCT for a radiomic model could be a valuable tool in predicting the clinical prognosis of AIS with large vessel occlusion.

## Introduction

AIS is a neurological emergency with high rates of disability and mortality (Regenhardt et al., 2018). According to statistics, ~25–35% of strokes manifest as large vessel occlusion, and this group is the main target for intravascular interventional therapy (Kidwell et al., 2013). However, the hyperdense areas on postoperative NCCT often confuse clinicians as to whether it was a hemorrhage or contrast agent and affect subsequent treatment and clinical prognosis.

The relationship between the hyperdense area and clinical outcomes remains uncertain. Some studies have shown that patients with the hyperdense area had a higher score on the modified Rankin Scale (mRS) score at discharge or 90 days than those without the hyperdense area (Payabvash et al., 2014, 2015; Rouchaud et al., 2014; Chen et al., 2019, 2020), while others indicated that it did not affect functional outcomes (Lummel et al., 2014; An et al., 2019). We would like to use a new machine learning tool that could obtain more information, including the area of the hyperdense area, the area of concomitant hypodense infarction, the histogram of CT value distribution, and the degree of brain parenchyma swelling to make a one-stop prediction of clinical outcomes.

Radiomics, as a new technology, transforms subjective visual interpretation into image data-driven objective evaluation in a non-invasive way. It can extract a large number of quantitative features, such as shape, intensity, and texture, from images and further reflect more biological information related to the disease (Lambin et al., 2012; Yip and Aerts, 2016; Avanzo et al., 2017). Radiomics has successfully demonstrated the potential for multiple applications in stroke, and the extracted features can be used to diagnose stroke lesions, predict early transformation, and assess the long-term prognosis after stroke onset (Chen et al., 2021; Jiang et al., 2021). Peter et al. (2017) identified six texture features from NCCT images that could differentiate ischemic lesions from their contralateral normal tissues. In addition, Tang et al. (2020) quantified the penumbra and core area from both the apparent diffusion coefficient and cerebral blood flow maps in patients with AIS (< 9 h) using radiomic analysis, and in the external dataset, the constructed radiomic nomogram could strongly predict favorable clinical outcomes at 7 days and 3 months. Clinically, NCCT is the first choice for AIS patients after intervention because it is efficient, non-invasive, and low in cost. Nevertheless, little is known about the relationship between the radiomics signatures based on NCCT after AIS intervention and the clinical prognosis.

Therefore, we aimed to develop a radiomics model to predict the clinical prognosis of AIS patients with interventional treatment. Then, the correlation between texture features and clinical outcome was further elucidated to identify potential biomarkers for clinical prognosis.

## Materials and methods

### Patients

This study was approved by the Ethics Committee of the Zhejiang Provincial People's Hospital. Due to the retrospective nature of the study, the patient's informed consent was waived. Patients' clinical data and NCCT data were obtained from routine clinical and radiological records. All patients with a clinically confirmed diagnosis of AIS who underwent interventional treatment from 1 January 2016 to 31 December 2020 were included. The inclusion criteria included (1) all patients who were diagnosed with AIS with large vessel occlusion (ICA isolated or in tandem with MCA) by preoperative one-stop head CT at admission, (2) intravascular intervention was carried out within the time window, (3) NCCT scan was performed immediately after patient intervention, and (4) complete clinical and imaging data could be obtained. The exclusion criteria included (1) patients with vascular malformation, intracranial hemorrhage, infection, or neoplastic lesions; (2) patients with a history of severe heart, lung, or kidney disease; and (3) postoperative NCCT images with obvious motion artifacts.

**Figure 1:**
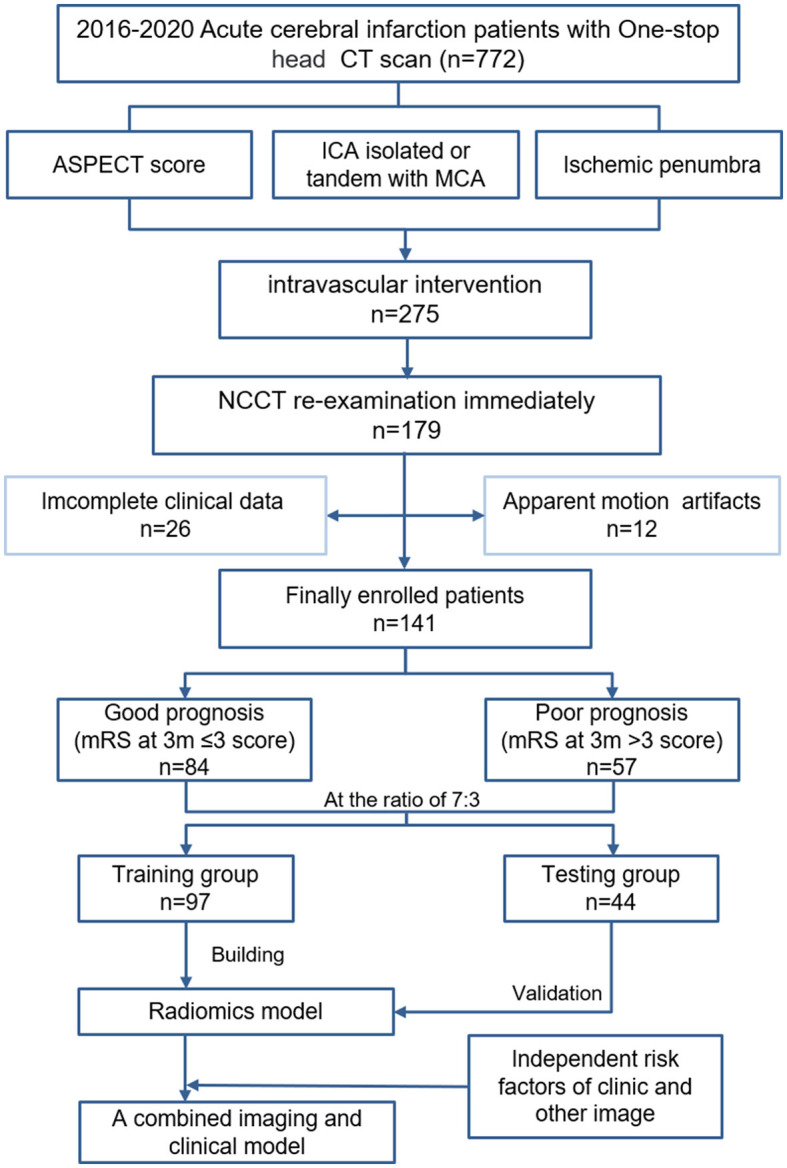
Flowchart of the recruitment path and research route used in this study.

The mRS and the National Institutes of Health Stroke Scale (NIHSS) were obtained from clinical records. The primary outcome measure was defined as whether the postoperative functional status was classified as a “good prognosis”, defined as a 90-day mRS 0-2, or “poor prognosis”, defined as a 90-day mRS 3-6, including severe disability and death. Finally, patients were divided into good prognosis (*n* = 84) and poor prognosis (*n* = 57) groups. At a ratio of 7:3, all patients were also divided into training (*n* = 97) and validation (*n* = 44) sets according to the subject serial number (Figure 1).

### CT data acquisition

NCCT scans after interventional treatment were performed using the Siemens Definition AS 128 CT. The routine head scan protocol: the tube voltage = 120 kV, the reference current = 400 mA, and the actual current can be adjusted by using the combined applications reduce exposure dose 4 dimensions (CARE dose 4D) technology: acquisition matrix = 512 × 512, rebuild FOV = 300 × 300 mm, layer thickness = 1 mm, and interslice gap = 0. The emergency head scan protocol: the tube voltage = 120 kV, the reference current = 400 mA, the actual current can be adjusted using the CARE dose 4D technology, acquisition matrix = 512 × 512, rebuild FOV = 300 × 300 mm, and pitch = 0:9 mm.

### Segmentation of region of interest

Based on the NCCT images after interventional treatment, 3D slicer software was used to segment 3D ROIs on the infarcted cerebral hemispheres. The detailed process is shown in Supplementary Figure S1.

### Image preprocessing and extraction of radiomics feature

**Figure 2:**
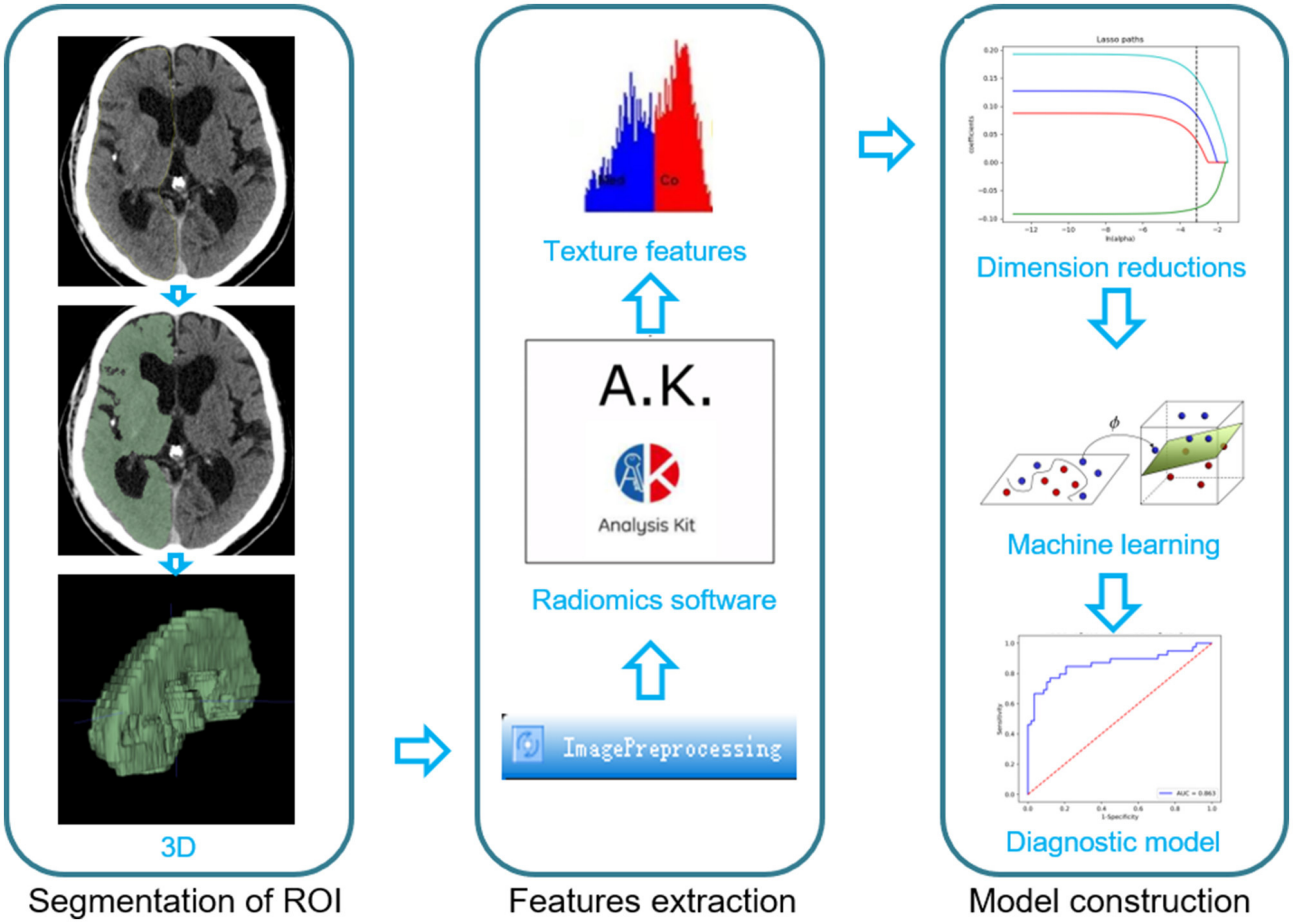
The main process for constructing the radiomics signature used in this study.

The images were preprocessed using AK software (Artificial Intelligence Kit V3.0.0.R, GE Healthcare), which included image interpolation, intensity normalization, and gray-level discretization as described previously. First, the image grayscale intensity level was discretized and normalized for noise reduction by downsampling each image into 25 bins. Given these fixed bin values and numbers, the grayscale range of the image was divided into equally spaced intervals. Next, we calculated 396 texture features, including histogram, formfactor, Haralick, run-length matrix (RLM), gray-level cooccurrence matrix (GLCM), and gray-level size zone matrix (GLSZM) with AK software. Prior to feature selection, all the extracted texture features were standardized. Dimension reduction was performed using analysis of variance and Mann–Whitney *U*-test and then we performed a correlation test to reduce data redundancy. Finally, the least absolute shrinkage and selection operator (LASSO) was used to further select significant features (Figure 2).

### Comparison of machine learning methods

A variety of machine learning algorithms, including, multivariate logistic regression (LR), Bayes, random forest, and decision tree classifiers, were undertaken to construct models based on the remaining features. The stability of each machine learning algorithm was quantified using the relative SD (RSD) and a bootstrap approach. For each classification method, we trained the model on a subsampled training cohort from the training set or the testing set and evaluated its performance on the remaining data using the area under the curve (AUC) of the receiver operating characteristic (ROC) curve. Subsampling of the training or testing set was performed 100 times using bootstrapping. RSD is the absolute value of the coefficient of variation and is often expressed as a percentage according to Equation: RSD = σAUC/μAUC → 100%, where σAUC and μAUC are the standard deviation and mean of the 100 AUC values, respectively. It should be noted that higher stability corresponds to lower RSD values. The calibration curve was used to describe the goodness-of-fit of radiomic models. Thereafter, we calculated the radiomic score (rad-score) for every patient in both the training and validation sets using the formula constructed in the training set.

### Establishment of the joint prediction model

In the training set, multivariable LR analysis was performed to select independent predictors of the clinical prognosis of AIS for each potential predictive variable, including age, gender, smoking, hypertension, diabetes, atrial fibrillation, use of anticoagulants, hyperlipidemia, the NIHSS at admission, the Alberta Stroke Program Early CT (ASPECT) score, bridging treatment, time of surgery, good revascularization, and rad-scores. Finally, the independent predictors from the training set were used to construct a joint prediction model using stepwise LR, and the data in the testing set were used to verify the performance of the joint model. Then, the ROC curves were used to visualize the experimental results, and the AUC was calculated to quantify the prediction performance.

### Statistical analysis

All statistical analyses were performed using SPSS (version 25.0) and R 3.5.1. The Kolmogorov–Smirnov test was used to test the normality of the data. Normally distributed data were evaluated using an independent sample *t*-test, whereas non-normally distributed data were evaluated using a Mann–Whitney *U*-test. The difference between categorical variables was tested with a chi-squared test. The correlations between mRS and optimal texture features were used in Spearman's analysis. Besides, the subjects were stratified into different subgroups using the median values of clinical factors and imaging biomarkers for correlation analysis. The Sankey diagram was used to show the relationship between these subgroups. A two-tailed *P*-value of < 0.05 indicated statistical significance.

## Results

### Patient clinical data

There were significant differences in the history of hypertension, the NIHSS score, the ASPECT score at admission, and the incidence of good revascularization between good and poor prognosis groups (all *p* < 0.05), as seen in Table 1. In the training set, there were significant differences in the history of hypertension, the NIHSS, and the ASPECT score at admission (all *p* < 0.05, Table 2).

**Table 1:** Basic characteristics of good and poor prognosis groups.

	**Good prognosis *n* = 84**	**Poor prognosis *n* = 57**	***P***
Age	70.12 ± 12.26	71.86 ± 11.11	0.392
Gender (male; %)	42 (50.0%)	36 (63.2%)	0.123
History of smoking	13 (15.5%)	12 (21.1%)	0.395
History of hypertension	57 (67.9%)	49 (86.0%)	0.015
History of diabetes	10 (11.9%)	11 (19.3%)	0.226
History of atrial fibrillation	39 (46.4%)	30 (52.6%)	0.470
History of anticoagulant use	17 (20.2%)	8 (14.0%)	0.344
History of hyperlipidemia	4 (4.8%)	3 (5.3%)	1.000
NIHSS at admission	18.75 ± 6.25	24.46 ± 6.80	0.000
ASPECT score (< 6 scores)	26 (31.0%)	38 (66.7%)	0.000
Bridging treatment	39 (46.4%)	21 (36.8%)	0.259
Time of operation (min)	328.43 ± 144.56	349.79 ± 143.42	0.389
Good revascularization (%)	76 (90.5%)	44 (77.2%)	0.030
mRS score at 3 m	2.37 ± 0.76	5.07 ± 0.84	0.000

HDL, high-density lipoprotein; LDL, low-density lipoprotein; APTT, activated partial thromboplastin time; PT, prothrombin time; HT, hemorrhagic transformation.

**Table 2:** Clinical and imaging information on training and testing sets.

	**Training set (*****n*** = **97)**			**Testing set (*****n*** = **44)**		
	**Good** ***N*** = **58**	**Poor** ***N*** = **39**	***p***	**Good** ***N*** = **26**	**Poor** ***N*** = **18**	***p***
Age	67.97 ± 11.99	70.62 ± 11.91	0.287	74.92 ± 11.69	74.56 ± 8.87	0.911
Gender	29 (50.0%)	25 (64.1%)	0.170	13 (50.0%)	11 (61.1%)	0.467
Smoking	11 (19.0%)	8 (20.5%)	0.851	2 (7.7%)	4 (22.2%)	0.208
Hypertension	40 (69.0%)	34 (87.2%)	0.039	17 (65.4%)	15 (83.3%)	0.303
Diabetes	4 (6.9%)	10 (25.6%)	0.017	6 (23.1%)	1 (5.6%)	0.211
Atrial fibrillation	25 (43.1%)	17 (43.6%)	0.962	14 (53.8%)	13 (72.2%)	0.346
Use of anticoagulants	7 (12.1%)	5 (12.8%)	0.912	10 (38.5%)	3 (16.7%)	0.182
Hyperlipidemia	2 (3.4%)	2 (5.1%)	1.000	2 (7.7%)	1 (5.6%)	1.000
The NIHSS at admission	18.88 ± 6.01	24.38 ± 6.70	< 0.01	18.46 ± 6.88	24.61 ± 7.20	0.007
The ASPECT at admission (< 6 score)	18 (31.0%)	27 (69.2%)	< 0.01	8 (30.8%)	11 (61.1%)	0.046
Bridging treatment	25 (43.1%)	12 (30.8%)	0.220	14 (53.8%)	9 (50.0%)	0.802
Time of operation	339.81 ± 155.52	368.51 ± 165.35	0.387	303.04 ± 115.17	309.22 ± 63.97	0.838
Good revascularization	55 (94.8%)	32 (82.1%)	0.084	21 (80.8%)	12 (66.7%)	0.288
mRS score at 3 m	2.38 ± 0.81	5.00 ± 0.86	< 0.01	2.35 ± 0.63	5.22 ± 0.81	< 0.01

HDL, high-density lipoprotein; LDL, low-density lipoprotein; APTT, activated partial thromboplastin time; PT, prothrombin time.

### Performance and stability of the machine learning methods

**Figure 3:**
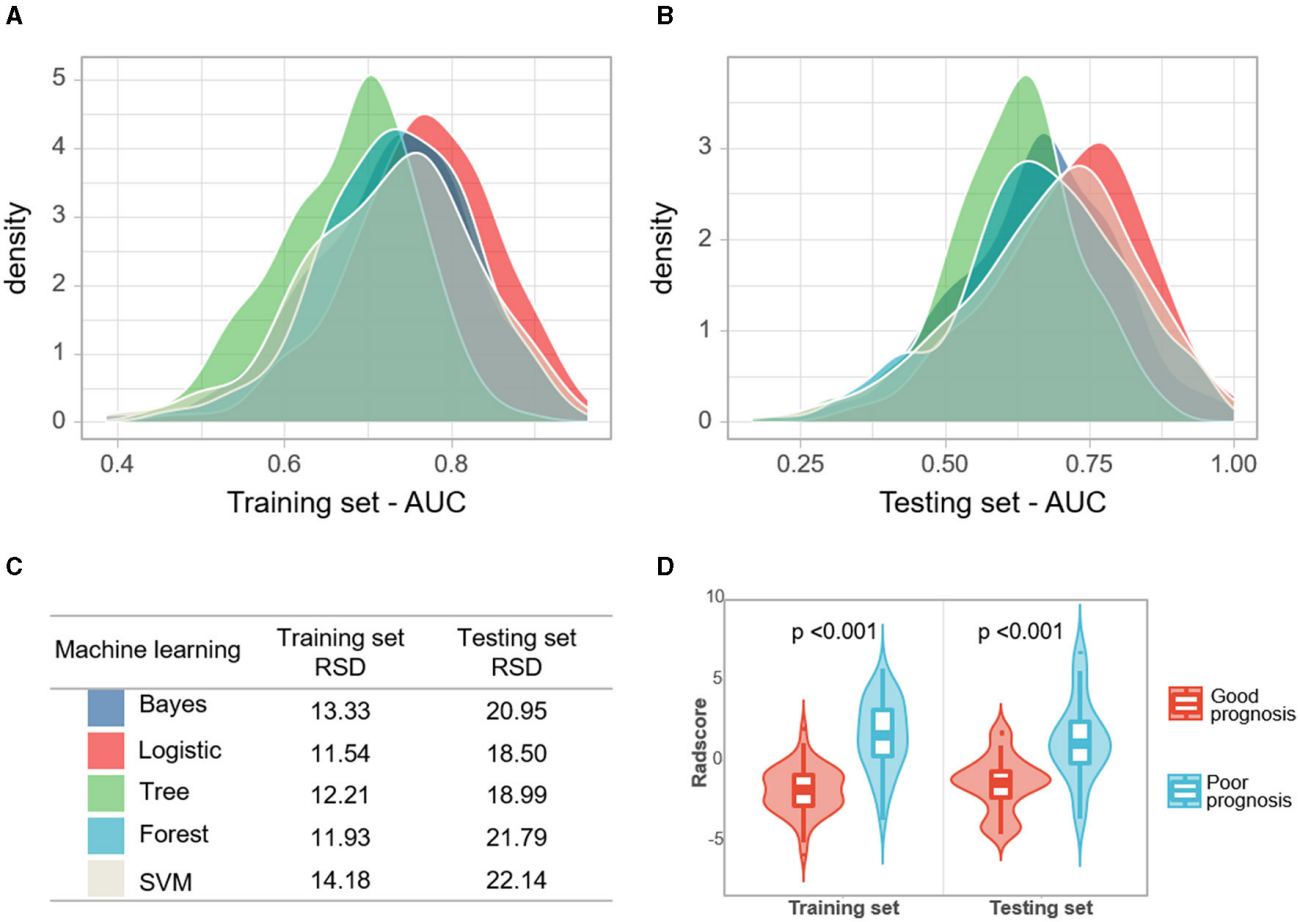
**(A, B)** Densities of AUCs from *in silico* validation of the machine learning models. **(C)** The relative standard deviation (RSD) of four algorithms, the lower RSD values correspond to the higher stability of the model. **(D)** Violin plots of rad-scores for the good and poor prognosis groups.

In the training set, the RSD values of the Bayes, LR, Tree, and Forest algorithms were 13.33, 11.54, 12.21, and 11.93, respectively. In the testing set, the RSD values for these models were 20.95, 18.50, 18.99, and 21.79, respectively. The LR algorithm showed better diagnostic performance and stability than the other machine learning algorithms in the training and test sets (Figure 3).

### Construction of the radiomics model

After standardization and dimension reduction, the four most valuable texture features were selected for the construction of the radiomics signature, including Range, Correlation_angle45_offset1, SurfaceVolumeRati, and VolumeMM. The main process of dimension reduction and the formula for the radiomics signature are provided in the Supplementary material. Based on the formula, the rad-scores were calculated, and it had favorable predictive efficacy in the training and testing cohorts (the AUC values were 0.863 and 0.840, respectively). The Hosmer–Lemeshow test revealed good goodness-of-fit of the radiomics model (all *P* > 0.05), and the calibration curves demonstrated good consistency in both the training and testing sets. The relevant results are shown in Supplementary Figure S2.

### Overall validation of the joint model

**Figure 4:**
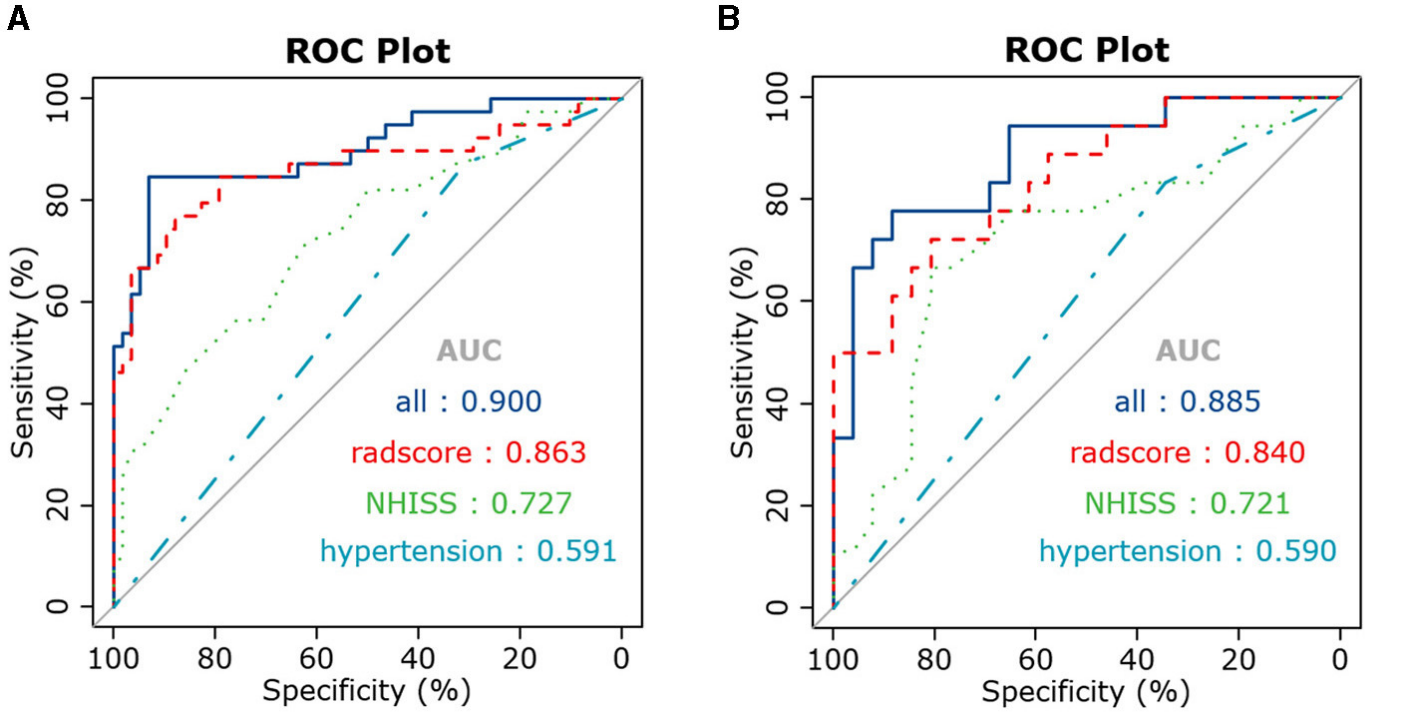
ROC curves of the joint model, radiomics signature, and clinical risk factors in the training **(A)** and testing **(B)** sets.

After stepwise LR, the NIHSS score at admission, hypertension, and rad-scores were used to build the joint model, as shown in Table 3. The AUCs of the joint model, rad-scores, NIHSS score, and hypertension were 0.900, 0.863, 0.727, and 0.591 in the training set, respectively. In the testing set, the AUCs of the joint model, rad-scores, NIHSS score, and hypertension were 0.885, 0.840, 0.721, and 0.590, respectively (Table 4; Figure 4).

**Table 3:** Stepwise logistic regression analysis predicting prognosis at 3 months.

	**Univariate logistic regression**		**Multivariate logistic regression**	
	**OR (95% CI)**	***P***	**OR (95% CI)**	***P***
Age	1.013 (0.984, 1.043)	0.390		
Gender	1.714 (0.862, 3.409)	0.124		
Smoking	1.456 (0.611, 3.473)	0.396		
Hypertension	2.901 (1.208, 6.970)	0.017	4.380 (1.351, 14.199)	0.014
Diabetes	1.770 (0.697, 4.494)	0.230		
Atrial fibrillation	1.282 (0.653, 2.515)	0.470		
Use of anticoagulants	0.643 (0.257, 1.611)	0.346		
Hyperlipidemia	1.111 (0.239, 5.163)	0.893		
The NIHSS at admission	1.142 (1.077, 1.211)	< 0.01	1.155 (1.067, 1.250)	< 0.01
The ASPECT at admission (< 6 score)	4.462 (2.173, 9.159)	< 0.01	/	/
Bridging treatment	0.673 (0.338, 1.339)	0.259		
Time of operation	1.001 (0.999, 1.003)	0.389		
Good revascularization	0.356 (0.137, 0.927)	0.034	/	/
Rad-score	2.611 (1.889, 3.609)	< 0.01	2.918 (1.969, 4.323)	< 0.01

HDL, high-density lipoprotein; LDL, low-density lipoprotein; APTT, activated partial thromboplastin time; PT, prothrombin time.

**Table 4:** Diagnostic performance of the joint model, radiomics signature, NIHSS score, and hypertension.

	**Training group**				**Testing group**			
	**All**	**Rad-score**	**NHISS**	**Hypertension**	**All**	**Rad-score**	**NHISS**	**Hypertension**
AUC	0.900	0.863	0.727	0.591	0.885	0.840	0.721	0.590
Sensitivity	0.846	0.769	0.718	0.872	0.778	0.722	0.667	0.833
Specificity	0.931	0.879	0.621	0.310	0.885	0.808	0.808	0.346
Negative predictive value	0.900	0.850	0.766	0.783	0.852	0.808	0.778	0.750
Positive predictive value	0.892	0.811	0.560	0.459	0.824	0.722	0.706	0.469
True positive rate	0.846	0.769	0.718	0.872	0.778	0.722	0.667	0.833
False positive rate	0.069	0.121	0.379	0.690	0.115	0.192	0.192	0.654
True negative rate	0.931	0.879	0.621	0.310	0.885	0.808	0.808	0.346
False negative rate	0.154	0.231	0.282	0.128	0.222	0.278	0.333	0.167
False discovery rate	0.108	0.189	0.440	0.541	0.176	0.278	0.294	0.531
Accuracy	0.897	0.835	0.660	0.536	0.841	0.773	0.750	0.545
Precision	0.892	0.811	0.560	0.459	0.824	0.722	0.706	0.469
Youden index	1.777	1.649	1.339	1.182	1.662	1.530	1.474	1.179

### Correlation between mRS and optimal texture features

**Figure 5:**
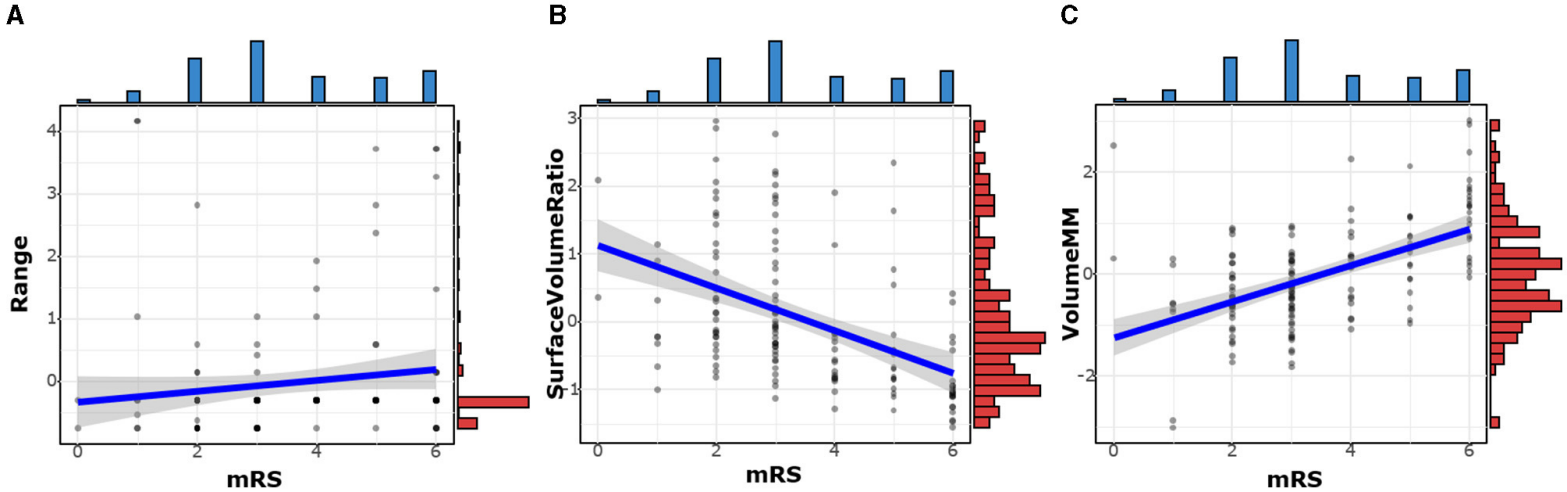
The correlation between mRS and optimal texture features. The mRS was positively correlated with Range **(A)** and Volume MM **(C)**. There was a negative correlation between mRS and Surface Volume Ratio **(B)**.

There was a negative correlation between mRS and surface volume ratio (*r* = −0.531, *p* < 0.001). However, the mRS was positively correlated with rad-scores (*r* = 0.570, *p* < 0.001), range (*r* = 192, *p* = 0.022), and volume MM (*r* = 0.510, *p* < 0.001), as shown in Table 5 and Figure 5.

**Table 5:** Correlation between mRS and optimal texture features.

	**mRS score at 3 m**	
	**Correlation index**	***p***
Rad-score	0.570	< 0.01
Range	0.192	0.022
Surface volume ratio	−0.531	< 0.01
Volume MM	0.510	< 0.01
Correlation_angle45_ offset1	0.097	0.255

**Figure 6:**
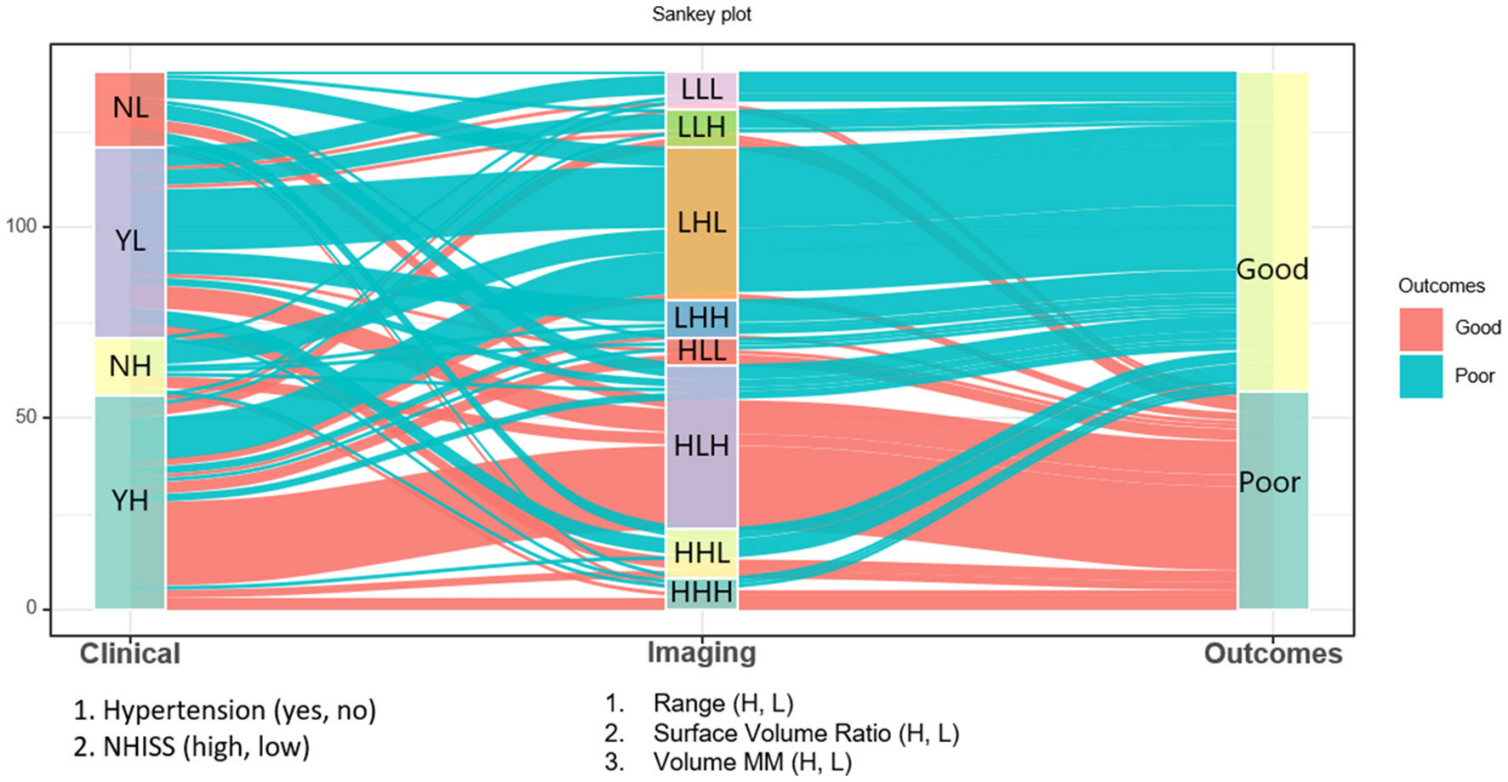
The Sankey diagram shows that most subjects with imaging LHL characteristics, which indicate a low *range*, high *surface volume ratio*, and low *volume MM*, have a good prognosis, whereas subjects with imaging HLH phenotypes, which denote high *range*, low *surface volume ratio*, and high *volume MM*, have a poorer prognosis. Besides, subjects with higher NIHSS scores and hypertension have a poor prognosis.

The relationships among clinical factors (NIHSS, hypertension), imaging biomarkers, and clinical outcomes were shown in the Sankey diagram (Figure 6). Each of the three imaging biomarkers that were correlated with mRS was divided into high (H) and low (L) based on their median values. The Sankey diagram shows that most subjects with imaging LHL characteristics, which indicate a low *range*, high *surface volume ratio*, and low *volume MM*, have a good prognosis, whereas subjects with imaging-HLH phenotype, which denotes high *range*, low *surface volume ratio*, and high *volume MM*, have a poorer prognosis. In addition, subjects with higher NIHSS scores and hypertension have a poor prognosis.

## Discussion

The innovation of our study was to develop and validate a new machine learning model based on reviewed NCCT after AIS intervention for predicting the clinical prognosis. According to our knowledge, this is the first study to construct integrative predictive modeling based on clinical data. Meanwhile, standard visual radiological and radiomic features of NCCT after AIS interventions were used to predict the clinical prognosis of patients, which showed good calibration and discriminatory ability in both the training and testing sets. In this study, we used NCCT and clinical data to predict the clinical prognosis of AIS after intervention, and the results demonstrated that comprehensive predictive modeling of rad-scores, the NIHSS score at admission, and history of hypertension with machine learning algorithms could accurately predict the clinical prognosis at 3 months for AIS patients after intervention. Moreover, our study found strong associations between radiographic markers (rad-scores and optimal textural features) and mRS at 3 months, which implied that biomarkers based on radiomic characterization of post-interventional NCCT could also be used to predict the severity of AIS outcome.

It is worth noting that our study showed a high correlation between optimal texture features based on NCCT and the mRS score. In overseas stroke clinical trials, the mRS is currently the most frequently used scale for assessing functional outcomes and can be a valid indicator of prognosis (McArthur et al., 2014). The surface volume ratio, as the name suggests, was the ratio of surface area to volume of ROIs. A lower value indicated a more compact (sphere-like) shape, a more swollen brain hemisphere, a higher mRS score, and a poorer prognosis. Similarly, a more swollen brain hemisphere indicated a larger number of ROIs and a poorer outcome, and our results also suggested that volume MM, a feature representing volume, was positively correlated with mRS scores. This was consistent with previous findings that cerebral infarct volume was highly correlated with brain damage and prognosis (Dastidar et al., 2000). In addition, the range of gray values in the ROI, had a slight positive correlation with the mRS score. We speculated that if there were both high density representing hemorrhage or contrast agent and low density representing infarction in the ROIs, the range of gray values would increase, and the corresponding clinical prognosis would be worse.

The NIHSS score and history of hypertension at admission were also independent predictors of the clinical prognosis of AIS after the intervention. Severe hypertension can lead to hemorrhagic transformation of the infarct, hypertensive encephalopathy, as well as cardiopulmonary and renal complications (Herpich and Rincon, 2020). A retrospective cohort study suggested that maintaining a range of 70–90 mmHg during endovascular therapy would improve functional outcome (Rasmussen et al., 2020). Anadani et al. (2019) showed that blood pressure control after revascularization was associated with an improved functional prognosis. The NIHSS score was also one of the central predictors that reliably predicted mRS-3 m. Brugnara et al. (2020)found that the most important parameter for predicting mRS 90 was the NIHSS score after 24 h (importance = 100%); this was consistent with our findings.

We acknowledge that the current study has several limitations. First, the retrospective nature of our study could not negate the risk of information and selection bias. However, the results obtained from this study enabled the development of a preliminary detection model. Second, the sample size of the model was relatively small; if we add further data in the follow-up, we will add more advanced machine learning methods, or even deep learning, to further improve our research. In the future, multi-center prospective studies with larger sample sizes would validate the accuracy of our model. Then, our inclusion of clinical data was largely limited to a simple clinical history and lacked detailed laboratory metrics, such as blood glucose fluctuations, blood pressure control, renal function, and cardiac conditions that may affect prognosis. Finally, owing to the difficulty of recognizing the real extent of acute cerebral stroke (ACS) after intervention by the naked eye, we designated the ipsilateral cerebral hemisphere region as the ROI.

In summary, our results showed that a predictive model had been identified by combining radiomic signatures, the NIHSS score at admission, and a history of hypertension. This model had the strongest power to individualize the prediction of future clinical outcomes for patients with AIS after interventional procedures. Instead of the classical mismatch concept, with advanced imaging technology, clinical guidance with radiomics methodology could add more value to the current clinical decision-making process. We expect that our model will be instrumental in the accurate prediction of AIS. Future prospective multi-center studies should aim to validate the efficiency of this model.

## Data Availability

The raw data supporting the conclusions of this article will be made available by the authors, without undue reservation.
