# Supplementary material for: Predicting the clinical prognosis of acute ischemic stroke using machine learning: an application of radiomic biomarkers on non-contrast CT after intravascular interventional treatment
Source: Front Neuroinform. 2024 Aug 22;18:1400702. doi: 10.3389/fninf.2024.1400702 (PMC11374607; doi:10.3389/fninf.2024.1400702)
Supplement: Supplementary file 1 [file Table_1.DOCX]

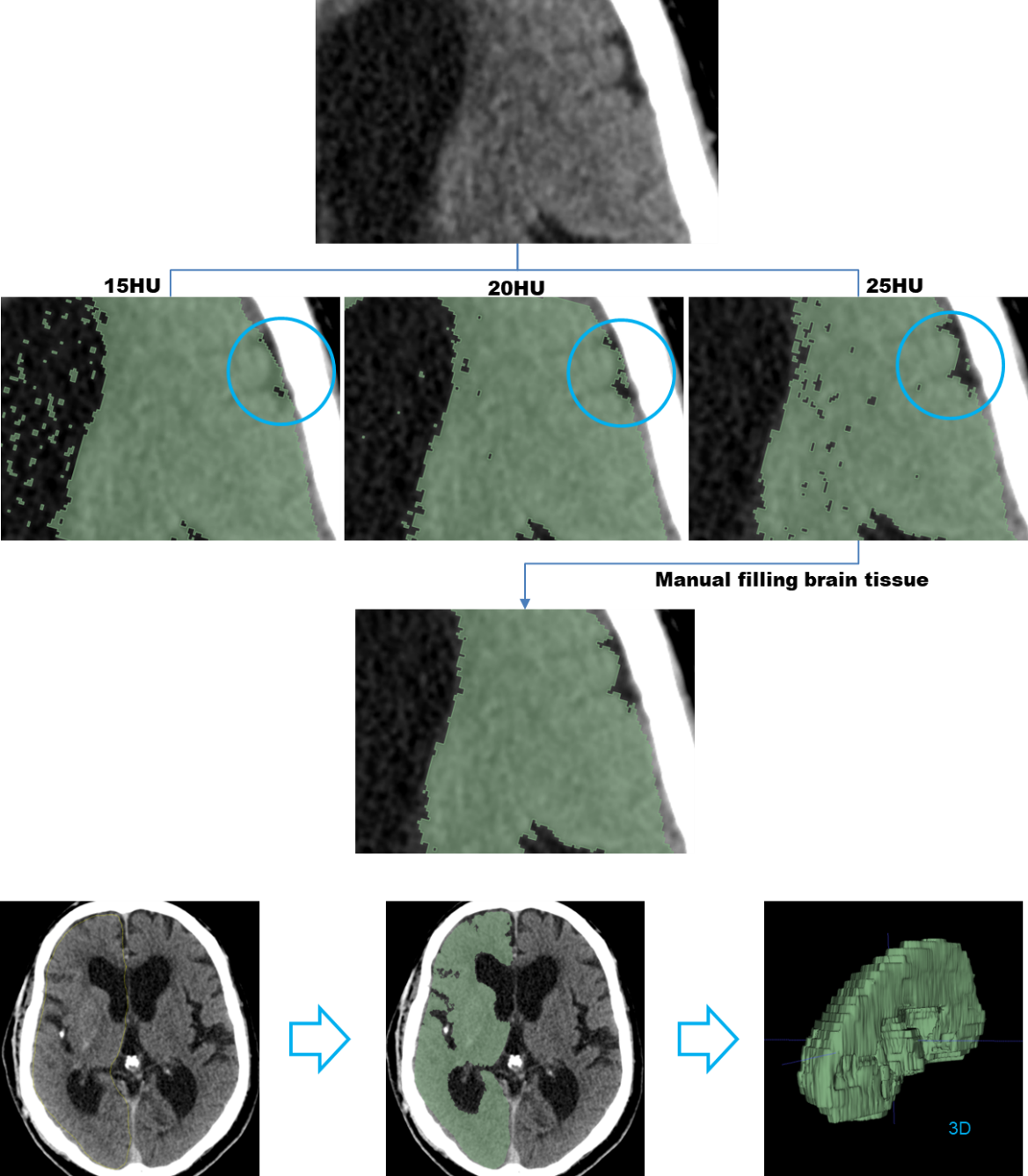


Fig s1. According to the CT value of cerebrospinal fluid was ranging from 0 to 20HU, the minimum thresholds for delineating ROIs were set as 15HU,20HU, and 25HU, respectively, in the pre-test. Finally, we found that the brain sulci would be best segmented when CT value was set at 25HU. Meanwhile, some brain tissue needed to be manually filled later. In practical work, we also make subtle adjustments to the threshold. Based on the NCCT images after interventional treatment, 3D slicer software was used to segment 3D-ROIs on the infarcted cerebral hemispheres.


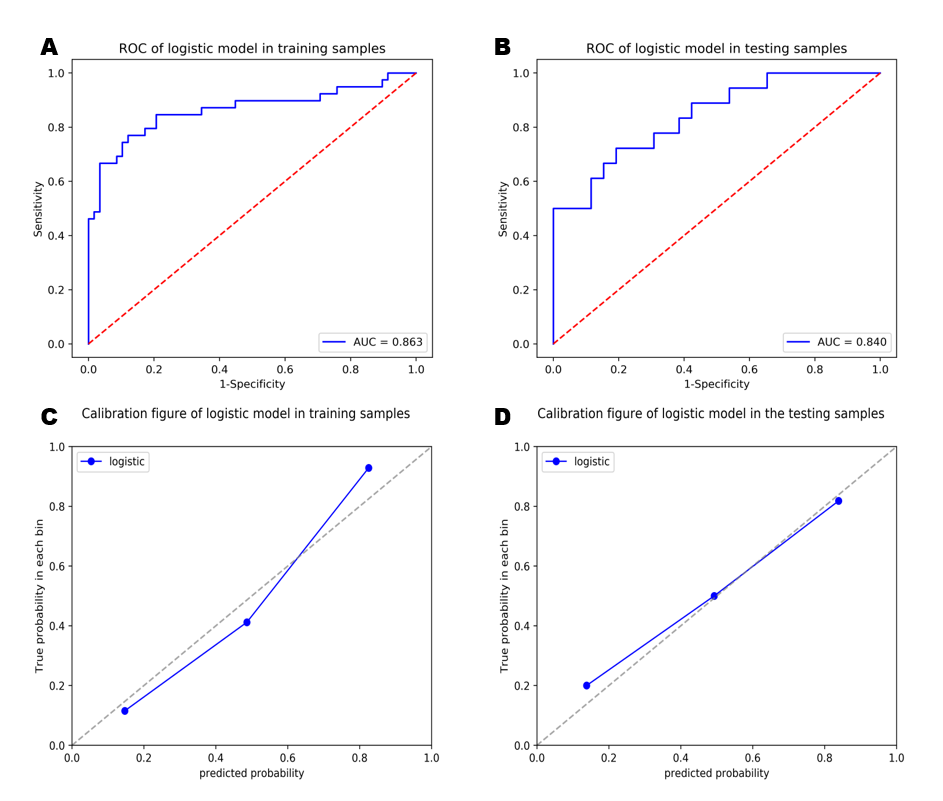

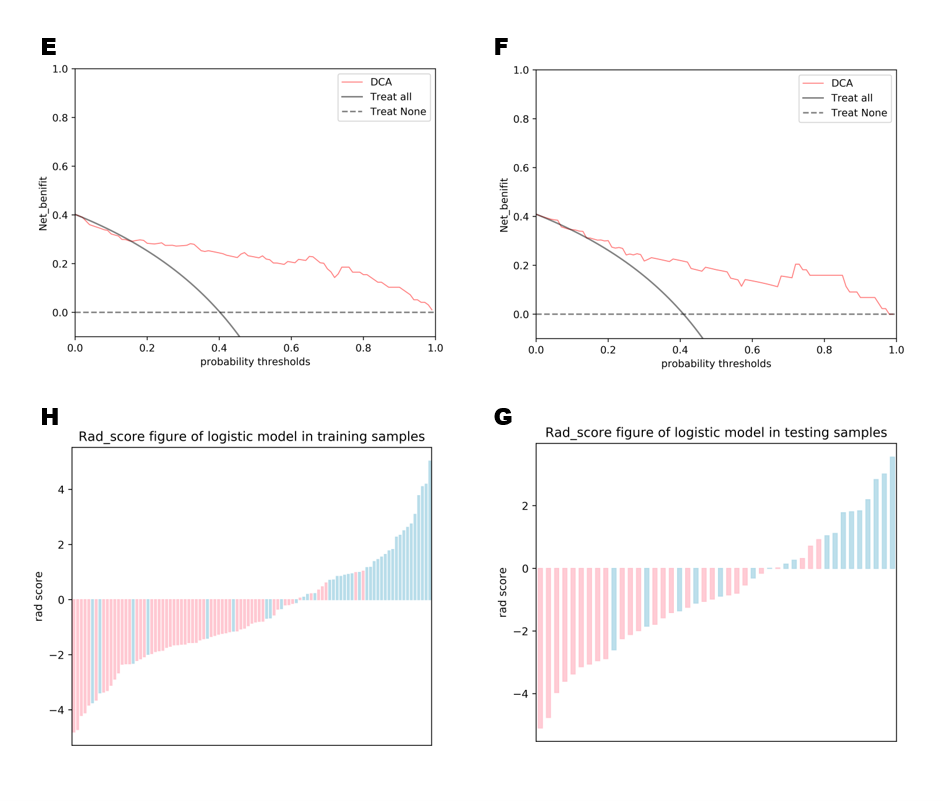


Fig s2. the AUC of the rad-scores in the training and testing cohorts were 0.863, 0.840, respectively. The Hosmer–Lemeshow test revealed good goodness-of-fit, and the calibration curves demonstrated good consistency.

**The main process of dimension reductions:**

# 1. The method for filling the missing data: Median

# 2. The method for standardizing the data: Standardization

# 3. The method for selecting features: Variance

parameters setted: {'threshold': 1.0}
num of remained features: 182

Heatmap of the model in the training samples:

Heatmap of the model in the testing samples:


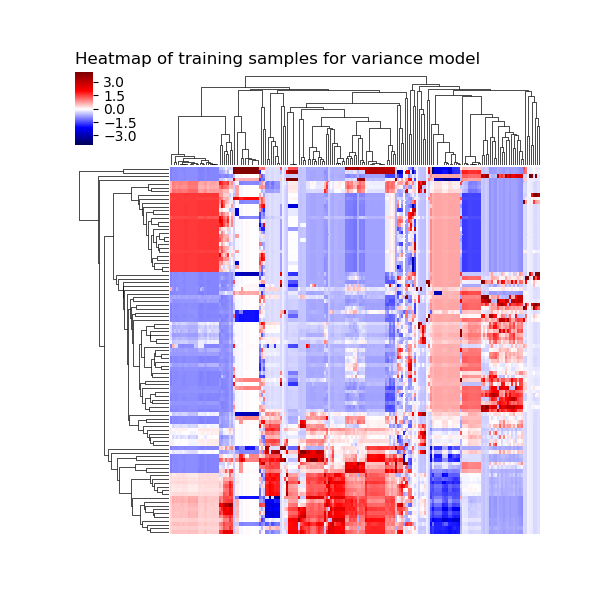

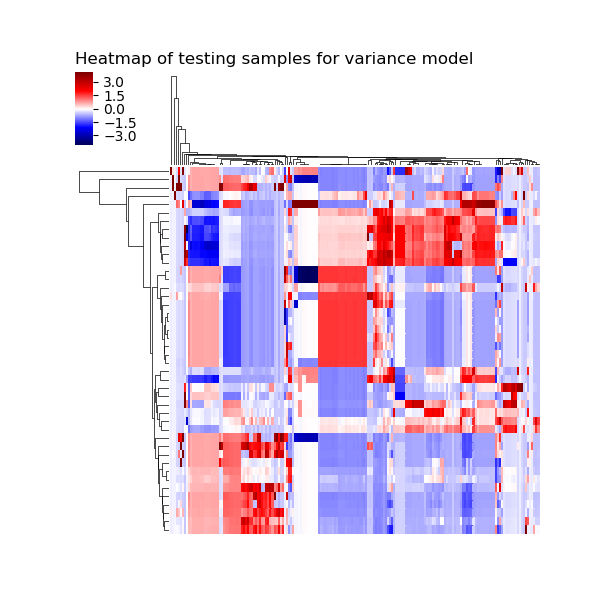


# 4. The method for selecting features: Correlation_xy

parameters setted: {'correlation': 0.1}
num of remained features: 92

Heatmap of the model in the training samples:

Heatmap of the model in the testing samples:


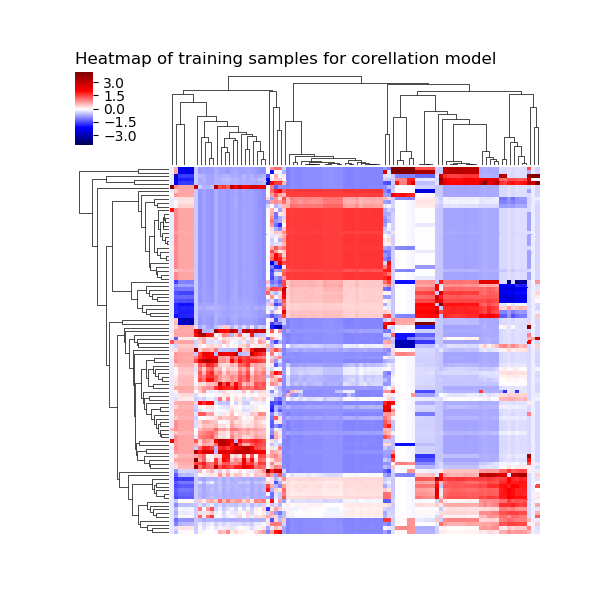

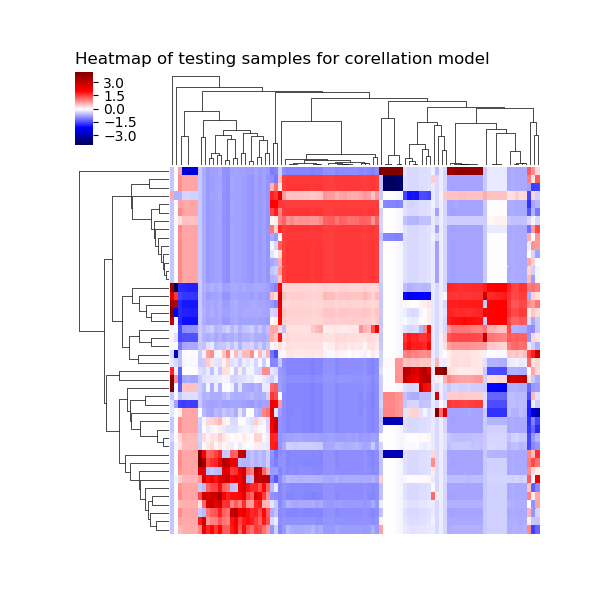


# 5. The method for selecting features: Correlation_xx

parameters setted: {'cutoff': 0.7}

num of remained features: 17

Heatmap of the model in the training samples:

Heatmap of the model in the testing samples:


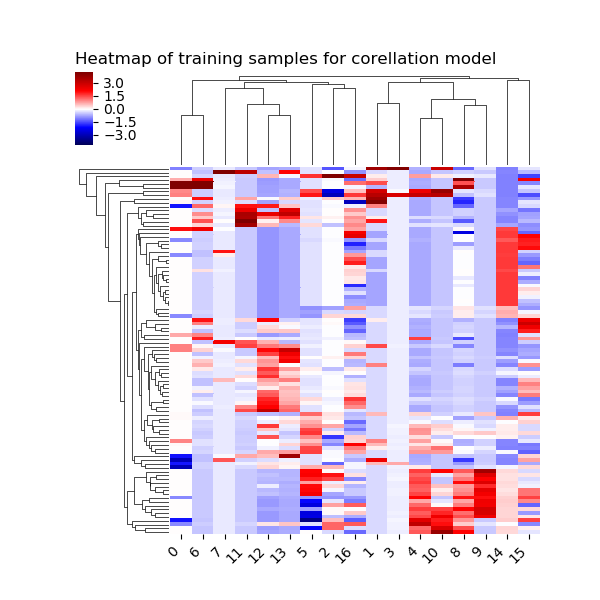

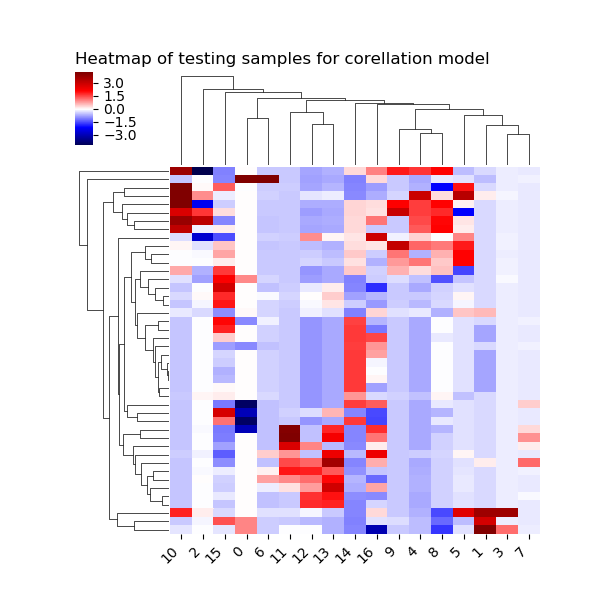


# 6. The method for selecting features: LASSON

parameters setted: {'alpha': 0.04443947970867156}
num of remained features: 4
remained features:
[['Range']
 ['Correlation_angle45_offset1']
 ['SurfaceVolumeRatio']
 ['VolumeMM']]

Heatmap of the model in the training samples:

Heatmap of the model in the testing samples:


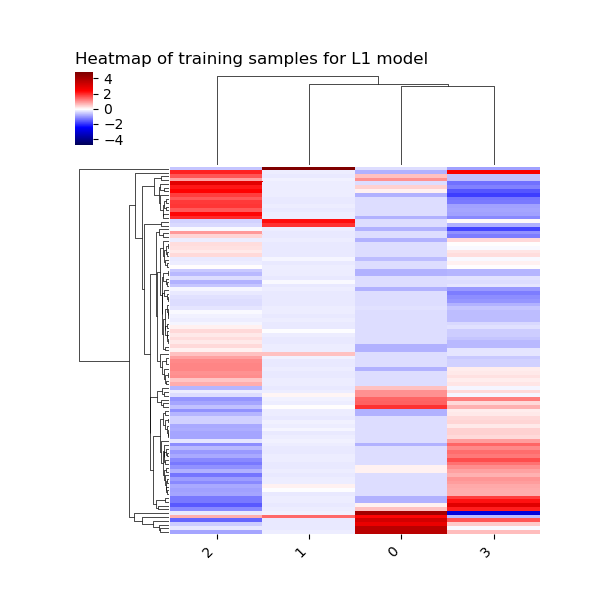

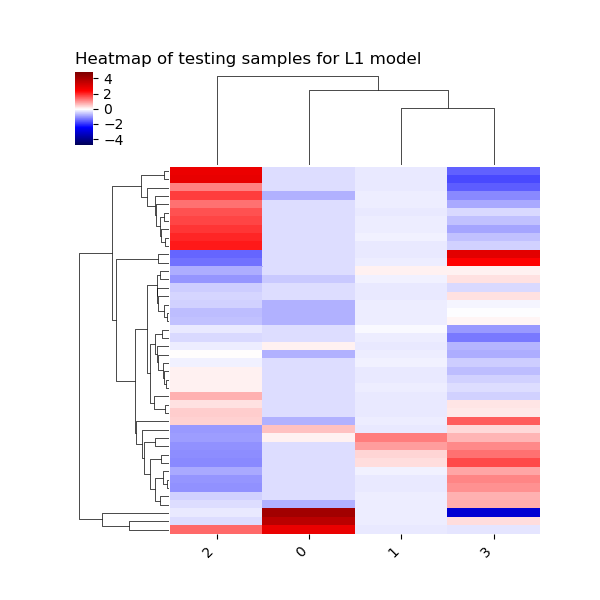


Correlation coefficient figure of the training samples

Correlation coefficient figure of the testing samples


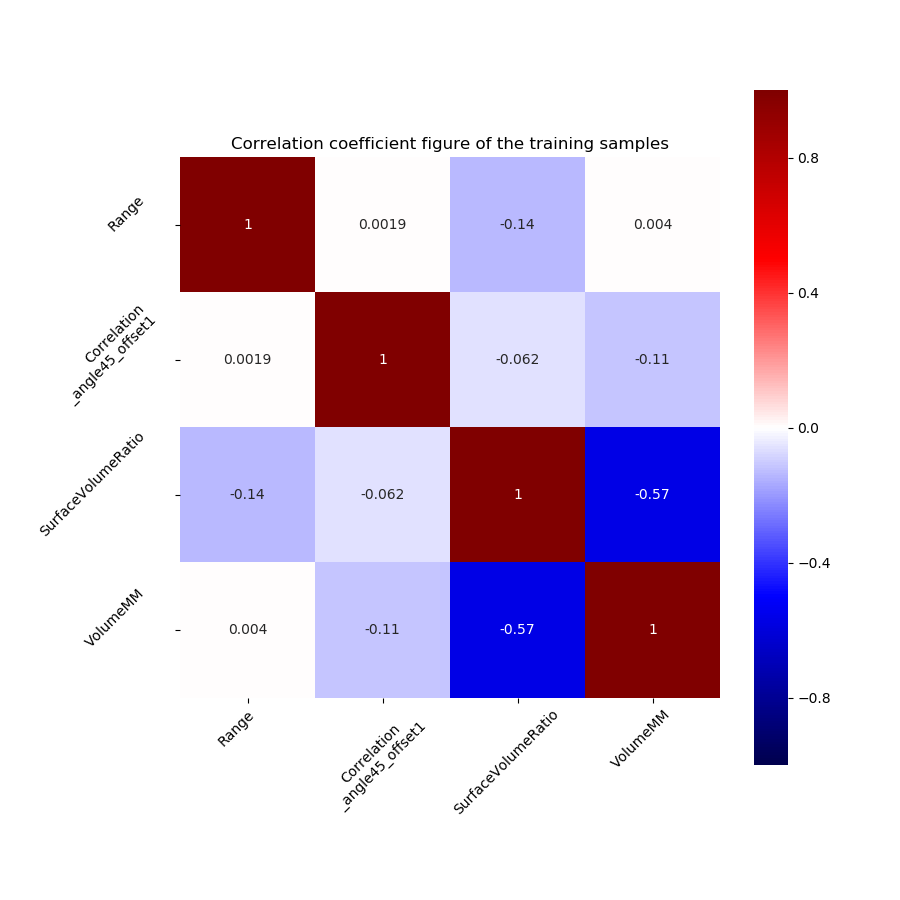

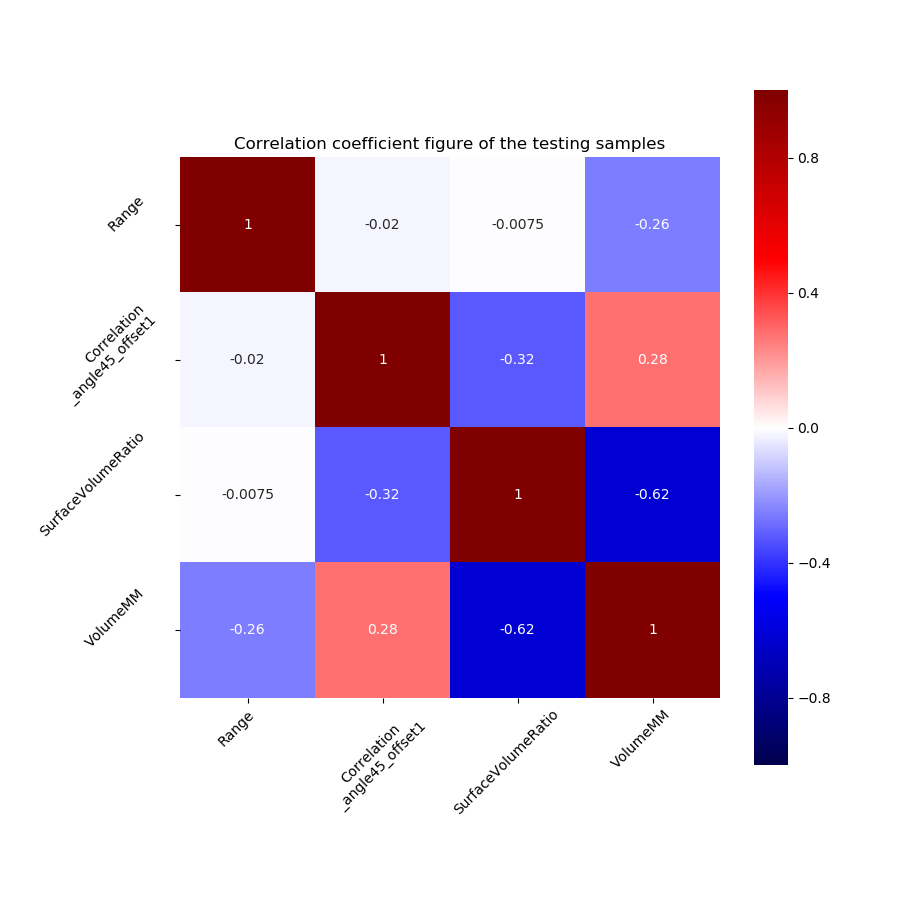


lasso path plot of the model in the training samples:


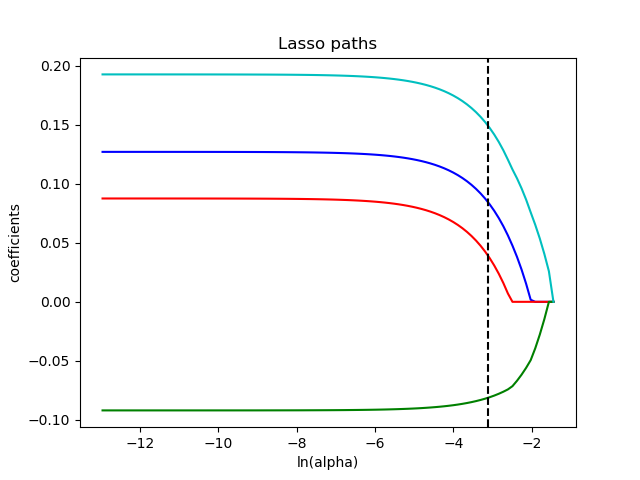


Mean square error on each fold for lasso model:


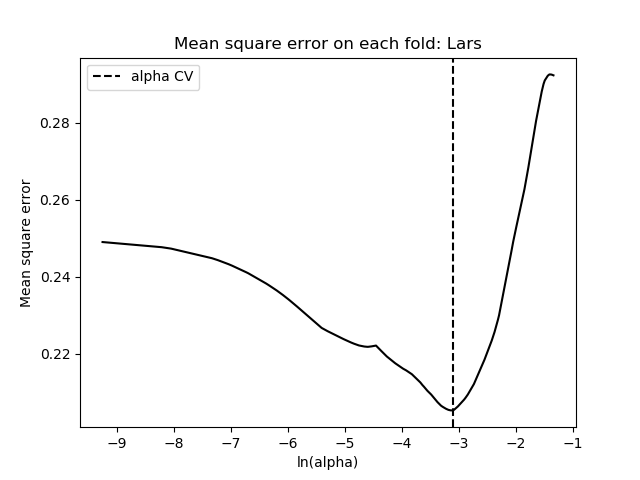


Rad-scores = -0.5700 +

0.8344 × Range +

0.6193 × Correlation_angle45_offset1 +

-0.7476 × SurfaceVolumeRatio +

1.1605 × VolumeMM
